# Evidence-based medicine: is it a bridge too far?

**DOI:** 10.1186/s12961-015-0057-0

**Published:** 2015-11-06

**Authors:** Ana Fernandez, Joachim Sturmberg, Sue Lukersmith, Rosamond Madden, Ghazal Torkfar, Ruth Colagiuri, Luis Salvador-Carulla

**Affiliations:** 1 Brain and Mind Centre, Faculty of Health Sciences, The University of Sydney, 94 Mallett Street, Camperdown, NSW 2050 Australia; 2 Discipline of General Practice, School of Medicine and Public Health, The University of Newcastle, Newcastle, Australia; 3 Centre for Disability Research and Policy, Faculty of Health Sciences, The University of Sydney, Sydney, Australia; 4 Menzies Centre for Health Policy, School of Public Health, Sydney Medical School, The University of Sydney, Sydney, Australia; 5 Centre for Disability Research and Policy-Brain and Mind Centre, Faculty of Health Sciences, The University of Sydney, Sydney, Australia

**Keywords:** Complexity of knowledge, Evidence-based medicine, Evidence-based practice, External validity, Framing, Generalizability, Internal validity, Randomized controlled trial

## Abstract

**Aims:**

This paper aims to describe the contextual factors that gave rise to evidence-based medicine (EBM), as well as its controversies and limitations in the current health context. Our analysis utilizes two frameworks: (1) a complex adaptive view of health that sees both health and healthcare as non-linear phenomena emerging from their different components; and (2) the unified approach to the philosophy of science that provides a new background for understanding the differences between the phases of discovery, corroboration, and implementation in science.

**Results:**

The need for standardization, the development of clinical epidemiology, concerns about the economic sustainability of health systems and increasing numbers of clinical trials, together with the increase in the computer’s ability to handle large amounts of data, have paved the way for the development of the EBM movement. It was quickly adopted on the basis of authoritative knowledge rather than evidence of its own capacity to improve the efficiency and equity of health systems. The main problem with the EBM approach is the restricted and simplistic approach to scientific knowledge, which prioritizes internal validity as the major quality of the studies to be included in clinical guidelines. As a corollary, the preferred method for generating evidence is the explanatory randomized controlled trial. This method can be useful in the phase of discovery but is inadequate in the field of implementation, which needs to incorporate additional information including expert knowledge, patients’ values and the context.

**Conclusion:**

EBM needs to move forward and perceive health and healthcare as a complex interaction, i.e. an interconnected, non-linear phenomenon that may be better analysed using a variety of complexity science techniques.

## Background

Over the past 20 years or more, the concept of evidence-based medicine (EBM) has increasingly been accepted as the gold standard for decision making in medical/health practice and policy.

EBM provides a standard procedure for using evidence in clinical decision making. It is framed as “*…the conscientious, explicit and judicious use of best evidence in making decisions about the care of individual* [sic] *patients. The practice of evidence-based medicine means integrating individual clinical experience with the best available external clinical evidence from systematic research*” [1]. Muir Gray regarded this definition as too doctor-centric and expanded it to emphasize the importance of the patient perspective and proposed that, “*…evidence based clinical practice is an approach to decision making in which the clinician uses the best scientific evidence available, in consultation with the patient, to decide upon the option which suits the patient best*.” [2]. In their respective papers, both Sacket and Gray described the stages of EBM decision making as (1) assessment and synthesis of external evidence using clinical epidemiology, systematic search and meta-analysis, and other techniques such as cost analysis and modelling; (2) use of probabilistic reasoning, taking into account, clinical expertise, and patients’ values and preferences. Remarkably, this broad but sensitive approach to rational clinical decision making was actually followed when applied to guideline development, but reduced the evidence in a skewed manner. Only evidence from explanatory randomized controlled trials (RCTs) was admitted as ‘reliable evidence’.

Whilst the value of EBM has been staunchly defended by its proponents, it has been widely criticized by many disciplines including clinical practice [3–8], epistemology [9–14], health sociology [15, 16], and implementation science [17]. Moreover, in recent years, previously supportive EBM researchers argue for a ‘renaissance’ of the movement that follows and applies their original broad principles and multidisciplinary values, specially regarding the components of EBM related to shared decisions with patients and to expert judgment, built of evidence and experience [18, 19]. The main argument is that, in spite of its benefits, EBM could have also had important negative consequences for healthcare delivery, policy and financing. Examples of this include (1) failing to manage complexity, the individual’s needs, and the person’s context and issues such as multi-morbidity; (2) the quantity of research studies and the variable quality, which has become impossible to manage and in some cases lack clinical significance; and (3) the medicalization of life, namely creating new diseases for non-specific complaints and the use of the evidence-based ‘quality markers’ to widely promote drugs and medical devices [20–22].

This paper contributes to the descriptive rational reconstruction of EBM by analysing its historical development and controversies [23], as well as its limitations in the current healthcare context. We approach this analysis from a complex adaptive systems science perspective with its focus on the relational interactions of health and healthcare variables [24] and the unified approach to the philosophy of science as suggested by Schurz [23]. A complex adaptive view of health as a balanced state between the person’s physical, social, emotional and cognitive experiences and its consequences for shaping complex adaptive healthcare and healthcare systems as highly responsive to the person’s unique needs as well as a complex adaptive understanding of medical knowledge have been described in detail elsewhere [25–27]. The unified approach to the philosophy of science provides a systematization of the basic assumptions of scientific knowledge and revises the role of values in science. It provides a new framework for understanding the differences between the phases of discovery, corroboration and implementation in science. Its importance for defining new areas of scientific knowledge and the role of different logic inferences in each phase have been reviewed elsewhere [28].

The present paper is structured in four sections: in the first, we review the origins, principles and actors who contributed to the rise of EBM, whilst in the second, we discuss why this movement evolved so rapidly and was so broadly accepted. The third describes a ‘restricted’ approach to EBM and its use in designing standard methods for developing practice guidelines, and finally, we comment upon the current challenges faced by the EBM movement in the context of systems thinking and implementation sciences.

### Where does EBM come from?

There were three factors at the beginning of the 20th century that predated the development of EBM, namely (1) the transformation of hospitals in the USA, from a shelter for the sick, to prestigious organizations, where medical care was based on scientific principles [29]; (2) the reform of medical education [30], and (3) the birth of clinical epidemiology [31]. The transformation of hospitals was accompanied by a process of standardization of healthcare provision through guidelines, which was also closely related to the efforts of the American Medical Association to establish its position as the reference accreditation body in medicine [29]. Standardization included the regulation of the medical profession, which ensured surgeons were well trained; the development of procedural standards in hospitals, which reduced variability and improved quality; and inclusion, for the first time, of the patient record file, allowing hospital managers to control what the physicians were doing [32]. As Timmermans and Berg suggested [29] the use of standards and guidelines, together with the emerging scientific knowledge and technologies enabled the growth of professional autonomy. However, standards and guidelines also became major triggers for the decline in clinical autonomy by the late 20th century [29].

The subject of clinical epidemiology was progressively introduced into medical programs based on the Enlightenment idea that progress was achievable through objectivity and rationality, so medicine has to be a science, not an art [29]. In 1968, McMaster University (Canada) was the first to offer an integrative ‘problem-based learning’ curriculum, combining the studies of basic sciences, clinical epidemiology and clinical medicine resulting from clinical problems [31, 33]. The ‘father’ of EBM, David Sackett, directed this department. The publication of a series of recommendations by the Canadian Task Force on Periodic Health Examination that was led by Sackett [34, 35] in 1979, underscored the rationale for using insights from clinical epidemiology to inform clinical practice. The findings supported recommendations to abandon routine annual check-ups in favour of selective approaches based on the patient’s age and sex. It was the first time that recommendations were made according to the ‘levels of evidence’ and exclusively based on ‘grading study designs’, i.e. RCTs provide good evidence (level I), cohort studies and case–control studies provide fair evidence (level II), and expert opinion arising from clinical experience provides poor evidence (level III). Unsurprisingly the same basis for grading treatment recommendations was applied from level A: to apply the intervention to level E: not to apply the intervention.

A prerequisite for the widespread adoption of EBM required clinicians to be more critical when appraising the scientific literature. In 1981, Sackett et al. [33] published a series of articles in the *Canadian Medical Association Journal* that explained the criteria for assessing the internal validity of study designs as RCTs providing the gold standard for treatment, cohort studies for diagnosis, and case–control studies for etiology or harm. However, as Zimmerman indicated [31], this simplification was one of the most important weaknesses of EBM. Indeed, the major resistance to EBM relates to the specification of the knowledge base of medicine as something rational/technical/linear/predictable rather than contingent/experiential/non-linear/unpredictable [16, 26, 36].

Managing the vast amount of research literature became possible with wider availability of computers, in particular the personal computer on the doctor’s desktop [37]. This enabled Iain Chalmers, director of the National Perinatal Epidemiology Unit in Oxford (United Kingdom) in the mid 1980s, to establish an electronic database of perinatal trials which made this information readily accessible to clinicians [38, 39]. The concepts and creation of electronic databases and increasing computing power facilitated the democratisation of knowledge management, something previously confined to only a few experts [40]. Some years later, The Cochrane Collaboration emerged as an organisation that systematically combed, reviewed and synthesised the vast amount of research literature to make it accessible to the clinician at the time of the patient consultation.

Another contextual factor to explain the development of the EBM was the rising concern about the sustainability of health systems during the 1970s. This concern resulted in the emergence of new disciplines, such as health economics, that influenced the development of major approaches to healthcare reform such as managerialism [41] and outcomes management [42], in addition to EBM. These three approaches focused on the ‘specific’ to achieve measurable objectives; continuous evaluation of performance against defined objectives, outputs and standards; and rationing of resources by effectiveness criteria to make the work of physicians more transparent through control and surveillance.

Closely related to the development of Health Maintenance Organizations was Outcomes Management (OM) in the United States, which adopted the principles of quality improvement to facilitate physicians’ autonomy and control of their clinical practice. OM follows four major principles [42]: (1) appropriateness, which relies on standards and guidelines; (2) routine outcome assessment based on routine and systematic measures of patients’ functioning and wellbeing, along with disease-specific clinical outcomes at appropriate time intervals; (3) the link to data mining to pool clinical and outcome data on a massive scale; and (4) a focus on dissemination and impact analysis to take into account the segment of the database most appropriate to the concerns of each decision maker. OM differs from EBM in its emphasis on ‘real data’ in contrast with EBM’s ‘experimental data’, while both OM and EBM aimed to empower clinicians to improve their clinical decision making capacity through the new tools on offer. This contrasts markedly with the view of managerialism, or neo-liberal approaches, where the power of decision making is shifted from clinicians to managers and auditors [41, 43].

The need for standardization, the development of clinical epidemiology, and concerns about the economic sustainability of health systems together with the increased capacity of computers to handle large amounts of data paved the way to the development of the EBM movement, officially founded in 1991 [33]. The publication of “Evidence-based medicine. A new approach to teaching the practice of medicine” by the Evidence-Based Working Group in *JAMA* [44] rapidly spread the concept and principles of EBM allowing the Evidence-Based Working Group to pronounce EBM to be a ‘new paradigm’. It would change the ‘old way’ of ‘solely’ practicing subjectivity-based medicine predicated on intuition, clinical experience and pathophysiological rationale with an objective approach based on ‘scientific’ evidence. Whilst they advocated the addition of evidence as a key consideration, they also clearly rejected the role of experts with ‘authoritative opinions’ as guiding clinical decision making.

### Why was EBM so widely accepted?

From 1992 until September 2015, the PubMed database revealed over 20,000 papers with ‘evidence-based’ in their title. Evidence-based practice guidelines are the norm for the majority of official agencies and professional organizations, and EBM approaches are at the core of today’s scientific thinking. The RCT is regarded as the fundamental research response underpinning the ‘perceived new paradigm’ of EBM for healthcare [45], and these ideas have now expanded far beyond the realm of medicine (consider, for instance, the debate if Conservation Science needs to include RCTs in the same way medicine does: http://blog.nature.org/science/2013/08/15/debate-randomized-control-trials-in-conservation/).

As Pope suggested [16], EBM evolved as a social movement that started with agitation (i.e. we need to change the current paradigm based on experience). It was crystalized by the shared experience of the group at McMaster University and the development of an enduring sense of purpose, disseminated in a series of position papers, declarations, and guidelines published in influential medical journals by key opinion leaders in clinical epidemiology. So, ironically, the adoption of EBM by the scientific community was not based on evidence but on authoritative knowledge, precisely the type of approach EBM was meant to replace, a point recently acknowledged by one of its key proponents, Sackett himself [46].

### Use of authoritative knowledge in EBM

We can identify three factors related to authoritative knowledge that could have played a major role in the success of EBM: reputation, the Matthew effect, and the invisible college.

The first ever paper on EBM written by an almost anonymous EBM Working Group ^a^ appeared in *JAMA* and provided the movement with instant grounds of credibility. Publishing under the authorship of a working group raised its status to that of an authoritative consensus paper. However, as Zimmerman suggested [31], the EBM Working Group used a language closer to a political manifesto, calling for far-reaching changes in the practice of medicine, in the process creating an ‘enterprise of scientific objectivity’. This working group, together with Drummond Rennie, deputy editor at *JAMA*, remained the main advocates of the EBM movement for the first critical years: out of 22 articles on EBM published in the first 3 years, 12 were published by *JAMA*, reflecting Rennie’s and JAMA’s remarkable commitment to the new approach [31, 46]. This new movement was not only sustained by *JAMA*, it also found the *British Medical Journal* to be its keen European supporter [1, 47].

Within 3 years, the movement was threatened by an equally prestigious journal, *The Lancet*, which took a critical position of EBM. In 1995, an anonymous editorial stating that although “*The Lancet applauds practice based on the best available evidence – bringing critically appraised news of such advances to the attention of clinicians is part of what peer-reviewed medical journals do – but we deplore attempts to foist evidence-based medicine on the profession as a discipline in itself”* [48]. *The Lancet* has since remained one of the most critical journals about the EBM movement. For instance, in 2005, it published a paper entitled “External validity of randomized controlled trials: To whom do the results of this trial apply?” criticizing the hierarchy of evidence as its focus is internal validity, neglecting the critical issue of external validity/generalizability of those results [49].

The reputations of the EBM movement’s key proponents and authors were well established. David Sackett and Iain Chalmers were renowned clinical epidemiologists and worked in highly regarded institutions – McMaster and Oxford University, respectively. Gordon Guyatt, as co-founder of the Medical Reform Group, a Canadian medical group composed of young doctors and nurses based in Toronto [31], notably agitated against the practice of medicine guided by senior doctors’ opinions. Personal experience gained under extreme conditions shaped the views of Archie Cochrane, a doctor and prisoner of war, and Iain Chalmers, a doctor in Gaza; they realized that in many cases new expensive treatments were no better than older ones [50]. From any point of view, the leading professionals were clearly well motivated but in practice their recommendations resulted in an over-simplified approach to ‘the clinical care of patients’.

A related reputational effect is gained from the Matthew effect [51] – raising the credibility of a viewpoint and an author group by excessive cross-citation amongst its proponents,^b^ a practice utilized by scientists since the 17th century. As a result, a group is highly likely to gain influence and power to set future research, practice and policy agendas (through grants, publications, conference presentations, etc.), made easier by the current state of publication policies and quality assessment procedures [52].

The extraordinary ability of the major EBM players to promote, implement and expand collaborative groups and networking resulted in what is known as an ‘invisible college’ [51]. The invisible college consists of a group of scientists or professionals who may live in separate locations but attend the same conferences, publish in the same journals, and invite each other to give keynote lectures to share the same ideas. An invisible college emerged from the collaboration between the groups at McMaster University and the Cochrane Collaboration. One could argue that the Cochrane Collaboration over time has morphed into a form of ‘visible college’. Indeed, the Cochrane Collaboration’s initiative of a series of small workshops started an international social network of EBM supporters.

### From a broad model to a narrow version of EBM

The historical and philosophical basis for EBM started with a broad health system’s perspective. In the 1930s, the then medical student, Cochrane, demanded on a protest placard that “*All effective treatment must be free*” [53, p. 1]. This call was about demonstrating a cost/benefit perspective, predicated on measuring “*the effect of a particular medical action in altering the natural history* [sic] *of a particular disease for the better*” [53, p. 2]. Cochrane argued that the RCT would remove bias and subjective opinion from managing disease, and indeed RCTs demonstrated important but limited gains in understanding therapeutic interventions. He clearly distinguished between ‘effectiveness’ and ‘efficiency’ and observed that, while the RCT as a scientific method could demonstrate ‘effectiveness’ in the trial population, this would not equate to greater ‘efficiency’ in healthcare, i.e. the same outcomes would generally not be achieved in routine practice due to the “*complexities within the health system*” [53, p. 2]. In addition, Cochrane was much more interested in the aspects of care ^c^rather than cure, alluding to the often neglected concern of ‘equality’ within the health system. As he stated: “*In particular I believe that cure is rare while the need for care is widespread* [sic]*, and that the pursuit of cure at all costs may restrict the supply of care, but the bias has at least been declared*” [53, p. 7].

The beginnings of the EBM approach were clearly focused on understanding the complexities of the ‘workings of the healthcare system’ and its relationship to making the ‘best possible decision’s for the care of patients’. However, these complexities have rapidly been reduced to a narrow focus on standardised and typically single disease management guidelines.

### Managing scientific knowledge for practice and the guideline development movement

One of the main objectives of EBM is to make large amounts of scientific knowledge more accessible, and developing clinical guidelines with recommendations to support clinical decisions seemed the obvious way to proceed.

Although clinical guidelines are useful they are also limiting if, for instance, they only draw on one source of information (i.e. the explanatory RCT). These guidelines will also restrain the freedom of professionals to use other sources of knowledge in their clinical decision making, like knowing patients’ preferences and clinical experiences [54]. Evidence-based guidelines for a specific area of practice are typically seen by clinicians as the penultimate and authoritative practice pathway, reinforced by adverse litigation and clinical review committee outcomes [29]. Consequently, many practitioners see clinical guidelines as the main threat to adapting clinical decisions to individual patients’ needs and contexts, i.e. interfering with their necessary clinical autonomy. Indeed, EBM supporters like JR Hampton, 32 years ago, asked for the death of clinical freedom as they saw ‘clinical judgment’ as the major obstacle to advancing medicine [55]; only recently they realized that clinical autonomy is needed so practitioners can use their ‘expert knowledge’ in the best interest of their patients [56].

The preoccupation with the quality of the studies used to develop clinical guidelines most likely explains the transformation of the broader EBM framework into its narrower RCT-driven form. The difficulties in translating the recommendations contained in EBM guidelines into practice and policy and the consecutive process of revision of the reductionist EBM approach to guidelines has been reviewed by others [49, 57, 58].

### ‘Grading’ knowledge

The initial problems with translating evidence-based guidelines into practice were attributed to the difficulties in properly rating the supporting body of research. Hence, a detailed analysis of the grading of RCTs was suggested [59]. The Grades of Recommendation, Assessment, Development, and Evaluation (GRADE) working group analyzed six grading systems used by 51 organizations and found, to its surprise, poor reliability in the assessment of the quality of studies. None of the systems were usable for all user groups (professionals, patients and policymakers). This study probably was the first major criticism of EBM by one of its leading groups, concluding that the systems for grading levels and strength of evidence had important shortcomings for proposing clinical recommendations [60]. The same group later recognized that the early systems of grading, which focused almost exclusively on randomized trials, were inadequate. The group realized that observational studies had features that could both decrease or increase the quality of the supporting body of research used for recommendations, and that high quality observational studies could contribute to better clinical decision making [61]. The factors considered when rating evidence include the overall quality of studies, the coherence of studies, uncertainties about the balance of benefits versus harms, and uncertainties in values and opportunity costs – reconnecting with Cochrane’s much broader vision.

Recognition of the need to separate assessment of quality of evidence from the strength of recommendations was a major step forward – “*high quality evidence doesn’t necessarily imply strong recommendations, and strong recommendations can arise from low quality evidence*” [62]. However, a new question arose: who should make these recommendations? Paradoxically, recommendations in clinical guidelines developed by ‘EBM experts’ use the opinion of their group of experts to work through and agree on the wording of their recommendations, grade the body of evidence, and utilize their values and background, i.e. apply their ‘prior expert knowledge’. However, how the information, expert opinion and contextual factors are balanced in these deliberations often remains unclear.

In recognition of the need for systematic and explicit approaches in the development and grading of recommendations, guideline development standards now require a summary of findings [63, 64] or evidence statements [65] for each recommendation and these are included in the guideline’s technical report.

### Various ways of knowing

The EBM community is starting to respond to criticism of limiting evidence and is attempting to incorporate and value other sources of knowledge, such as observational studies and the findings of qualitative research. Nevertheless, the fundamental flaws inherent in the grading systems remain such as the assumption that: study designs and quality can be arranged in a systematic but simplistic linear structure, when, in reality, the use of different and highly relevant information such as patient preferences, and applicability to local or practice contexts would lead to completely different grading systems [66]; and that studies graded according to epidemiological principles will diminish the risk of bias. This criterion may make sense in the discovery of new knowledge but is not the key consideration if the main aim is to implement research into local practice.

It is important to consider that scientific knowledge is divided in three major areas: discovery, corroboration and implementation, and that the information of one domain cannot be directly applied to the others [23, 28]. The value-neutrality principle that guides discovery and corroboration requires further – and complex – clarification in the implementation phase. According to Schurz [23], the value-neutrality requirement “*implies that the scientist separates her scientific knowledge from fundamental value assumptions which she assumes in means-end inferences*”. Means-end inferences and abduction are used in EBM guideline development without an adequate formalization of their contribution to the construction of the guideline recommendations [28]. The roots of this philosophical debate are far beyond the scope of this paper and, unfortunately, the philosophy of science principles necessary to support the underpinnings of EBM have as yet not been properly explored.

In the fields of discovery and corroboration the important criterion is internal validity (observed variation can be interpreted as a causal relationship, therefore, the study design needs to guarantee that the risk of bias is low). In the field of implementation, the important criterion is the degree of external validity of the results applicability to the local context and acceptability of the intervention/s to the patient. External validity is important as it means that the results can be generalized to different persons, settings and times. There is an inverse relationship between internal and external validity. If the final purpose of EBM was to improve the health of real people in real settings, external validity should be emphasized and strengthened. It is not only crucial to know if a treatment is effective in controlled situations (i.e. internal validity), but also that it is going to be effective in the real world (i.e. external validity). While the grading systems for developing clinical guidelines used by EBM are systematic and reliable, they often prioritize internal validity and therefore are not ‘fit for purpose’. The emphasis on internal validity has contributed to the failure of EBM, as recommendations – being based on experimental designs where variables and confounders are controlled (RCTs) – often fail to be translatable into practice because the research context does not reflect real world clinical practice/reality [67, 68].

The EBM and guideline communities have also recognized the limited implementation of guidelines jeopardized by their current static and unfriendly structure [18, 54, 69]. New proposals, such as the development of dynamic wiki-based clinical guidelines, might eventually resolve this problem and enable the participation of all stakeholders (e.g. patients, clinicians and decision makers), in a collaborative effort that may result in greater transparency and acceptability [70, 71].

The leap from discovery/corroboration to implementation was partly reflected in the criticism made in *The Lancet* in 2005 [49] and in the position of other EBM experts such as ER Epstein, who developed the disease management approach, superseded by Wagner’s chronic/integrated care model [71]. Even though he adhered to EBM, Epstein’s vision of health knowledge was clearly beyond RCTs and much closer to Paul Ellwood’s OM approach. He considered EBM as one of several tools to improve quality of care: “*The new paradigm is population-based risk and disease assessment, systems of disease prevention and health promotion, community-based intervention and provider contacts within a framework of automated information, evidence-based medicine, and defined protocols of care, with explicit collection of outcomes information*” [72]. Epstein and Sherwood, although subscribing to the gold-standard of RCTs, mention the difficulty of using them as the main source of information in outcome management/implementation and mention ‘prospective effectiveness trials’ as the alternative to RCTs [72, 73].

### The limitation of RCTs to assess real world outcomes

A more fundamental question would be, can real world outcomes be achieved/evaluated with randomized controlled trials? In short, the answer is no, if we only use explanatory randomized trials as preferred by its proponents. However, pragmatic controlled trials that, by definition, are conducted under usual conditions offering practitioners considerable freedom in deciding how to apply the intervention to be tested, are not obtrusive (i.e. there is no special effort to improve compliance by patients or practitioners), and use administrative databases for the detection of outcomes, can offer a valid alternative. While explanatory RCTs will be linked to discovery and corroboration and will aspire to removing variability, pragmatic controlled trials (even including randomization) fit in the area of implementation and embrace variability as the norm [74, 75]. They take into account the local context and are mostly valued when driven by theory and complemented by other sources of knowledge [76].

### In conclusion: the challenges facing EBM

Most likely, EBM grew too fast to effectively incorporate its original propositions: evidence, expert knowledge, and patients’ preferences [1]. The reliance of EBM on the RCT was useful for acute (mostly single disease) conditions treated with simple interventions, but this approach is not suitable in the current epidemiological context characterized by chronicity and multimorbidity in complex health systems. In particular, EBM has largely disregarded the importance of social determinants of health and local context – hence the nicknames ‘cookbook approach’ or ‘MacDonaldization’ of medicine [29, 77]) – and its real impact on the ‘effectiveness’ and ‘efficiency’ of healthcare on the ‘equality’ of needed healthcare services.

As an a priori, evidence is context sensitive, and therefore to some extent tacit [78], and both global and local evidence need to be combined in the development of usable recommendations for clinical decision making [79]. Local evidence includes the presence of modifying factors in the specific settings, magnitude of needs (prevalence, baseline risk or status), patient values, costs (to the patient and the system), and the availability of resources in the system [80]. This local evidence needs to be combined with ‘expert knowledge’, which should be differentiated from ‘expert opinion’ and valued in a different way. By ‘expert knowledge’ we mean the implicit knowledge that professionals have that helps them to better understand the local conditions. It is based on data (their accumulated experiences) and thus different to simple opinions or feelings about something [81, 82]. There is on-going debate of the relevance of ‘colloquial evidence’ in the development of guidelines [83]. This reflects a worrying lack of a basic understanding by authors and reviewers of the fundamentals of scientific knowledge and the differences between expert knowledge and evidence.

There is an imperative to explore and then learn from other disciplines on how to use research evidence and incorporate it with local context and expert knowledge to achieve best possible patient outcomes. For example, in other areas of science, e.g. conservation science and artificial intelligence, expert knowledge is routinely incorporated in the analysis. Expert-Based Collaborative Analysis is a systematic procedure to incorporate expert knowledge into data analysis; such an approach has been proven to be useful when dealing with complex issues and can be seen as a powerful tool in the current health context characterized by an increase in the number of patients with multiple conditions, resulting from heterogeneous genomic/pathophysiological pathways and diverse personal needs [81].

In future, the inclusion of ‘expert knowledge’ in the analysis of research data might produce more usable evidence for clinical decision-making. In this sense, EBM needs to go beyond the sole use of the RCT and acknowledge that scientific knowledge is multidimensional and cannot be arranged in only one hierarchical system. Knowledge coming from studies using different methodological approaches is complementary [28]. Hence, to have a complete picture, information coming from explanatory RCTs has to be complemented and contrasted with information coming from pragmatic RCTs evaluating effectiveness in routine practice. This implies some loss of ‘internal validity’ and an increase in the uncertainty of the results, but ‘gains in representativeness’.

The most important challenge facing the EBM-movement is the provision of a detailed description of its methods for scientific reasoning. This requires an analysis of its taxonomic principles, including formal definitions of ‘scientific knowledge’, ‘evidence’, and ‘decision making’ in health, as well as the different types of logic inferences used in the scientific reasoning process [28]. As others have highlighted, we believe that this academic exercise is crucial to clarify the confusion between ‘good’ evidence [84] and scientific ‘truth’. Apart from systems thinking [85, 86], healthcare researchers, clinicians and policymakers could benefit from greater knowledge of the philosophy of science to design and interpret research, and their use in guiding decision making processes – beyond the classical experimental/deductive approach favoured by the EBM movement [18, 23, 28, 87].

It should also be highlighted that health systems research involves different disciplines (including social ones) with different perspectives, epistemologies, and ways of conceptualizing and conducting research. Health systems research, as intimated by Cochrane, is broader than identifying ‘clinical effectiveness’ – ‘efficiency’ and ‘equality’ are equally important considerations for achieving successful implementation of health system improvement; therefore, all stakeholders’ fundamental value assumptions should be explicit.

### Ethics

Ethics approval was not required as this manuscript is a narrative review of published papers.

## Endnotes

^a^They signed as a group but in a footnote they included their names.

^b^It refers to the fact that even though the majority of papers have little impact on the scientific community (the hath not), a small number of them attracts the attention of the community and receive a lot of citations (the hath abundance).

^c^As an aside, Cochrane’s observations working as a doctor in a prisoners of war camp provided him with the empirical evidence of the great benefits of care, its scientific basis has been untangled by the study of psychoneuroimmunology.

## Abbreviations

EBM: Evidence-based medicine
OM: Outcomes management
RCTs: Randomized controlled trials
